# Shifting Attention within Memory Representations Involves Early Visual Areas

**DOI:** 10.1371/journal.pone.0035528

**Published:** 2012-04-25

**Authors:** Jaap Munneke, Artem V. Belopolsky, Jan Theeuwes

**Affiliations:** 1 Department of Cognitive Psychology, VU University Amsterdam, Amsterdam, The Netherlands; 2 Center for Mind/Brain Sciences (CIMeC), University of Trento, Trento, Italy; University of Leuven, Belgium

## Abstract

Prior studies have shown that spatial attention modulates early visual cortex retinotopically, resulting in enhanced processing of external perceptual representations. However, it is not clear whether the same visual areas are modulated when attention is focused on, and shifted within a working memory representation. In the current fMRI study participants were asked to memorize an array containing four stimuli. After a delay, participants were presented with a verbal cue instructing them to actively maintain the location of one of the stimuli in working memory. Additionally, on a number of trials a second verbal cue instructed participants to switch attention to the location of another stimulus within the memorized representation. Results of the study showed that changes in the BOLD pattern closely followed the locus of attention within the working memory representation. A decrease in BOLD-activity (V1–V3) was observed at ROIs coding a memory location when participants switched away from this location, whereas an increase was observed when participants switched towards this location. Continuous increased activity was obtained at the memorized location when participants did not switch. This study shows that shifting attention within memory representations activates the earliest parts of visual cortex (including V1) in a retinotopic fashion. We conclude that even in the absence of visual stimulation, early visual areas support shifting of attention within memorized representations, similar to when attention is shifted in the outside world. The relationship between visual working memory and visual mental imagery is discussed in light of the current findings.

## Introduction

Spatial attention is the ability to dynamically allocate processing resources to a limited part of the visual environment [1]–[3]. Prior studies have shown that in order to facilitate the selection of relevant visual information needed for the observer's current goals, attention is used to bias perceptual processing of external visual representations of objects or locations. This model of “sensory gain” has been motivated by studies that showed increased neural activity in visual areas as a direct result of focused attention. Modulation of neural activity in visual areas as a result of the allocation of attention to visual information has been observed in monkeys, using single-cell recording [4], [5] as well as in healthy humans using PET [6]–[8] and fRMI [9]–[11]. Moreover, allocating attention to regions in the visual field has shown to modulate neural activity in both striate and extrastriate cortex in a retinotopic fashion [12]–[14]. Thus, when attention is allocated to objects or locations in the visual field, the perceptual representation of this information is enhanced as indexed by increased neural activity in visual areas that code the attended information.

Although a large body of evidence has emphasized the role of attention in the perception and selection of visual information presented to the visual system, less is known about how attention influences internal representations of visual information stored in working memory. Working memory is the ability to actively maintain a representation of visual information in mind, to the extent that we can utilize this stored visual information even when this information is no longer present to the visual system [15]. A functional subset of this system, known as visual-spatial working memory (henceforth called “spatial working memory”) deals with the maintenance of memory representations of location-specific information, such as remembering where a certain object was presented in a scene (for a review see [16]).

The influence of attention on spatial working memory representations has been addressed in a number of studies [17]–[21]. To address this issue, Griffin and Nobre [20] used a location cueing task in which on a particular trial, participants were cued with 80% validity which item in an array of stimuli was the target. The cue could appear prior to the onset of the stimulus array (pre-cue) or after the offset of the array (retro-cue). At the end of the trial, participants indicated whether a presented probe had been present or absent in the stimulus array. By using a retro-cueing procedure, the benefits of cueing a location within an internal representation stored in working memory could be studied and could be compared to a condition in which similar visual information was pre-cued. Results showed a validity effect: participants responded faster and more accurate to a target that was validly cued than to a target that was invalidly cued. Importantly, no difference in performance was observed between trials in which a location was either pre-cued or retro-cued. Both trial types were observed to yield faster response times compared to neutral cues in which no spatial information was provided to the participants. This study clearly shows that items held in working memory can be individually singled-out and modulated by spatial attention. It was concluded that cueing a location in a working memory representation is just as beneficial as cueing an actual perceptual representation in the outside world. Therefore it appears that attention functions in a similar fashion for the selection of items in an internal representation (i.e., working memory) as for the selection of items in the outside world.

In a similar line of research, Belopolsky and Theeuwes [17] performed an eye movement study in which participants were instructed to remember the location of two circles presented in the left and right visual field. After an initial delay period during which the circles were removed from the screen, a cue indicated which of the two stimuli should be maintained in working memory for the remainder of the trial. After a second delay period, participants were instructed to make a rapid saccade either up or down along the vertical meridian. The results of the study showed curvature away from the memorized location compared to a non-memorized location. Curvature away is thought to reflect inhibition of oculomotor programs evoked by attended locations [22], again showing that selection of individual stimuli can take place within a working memory representation.

Further evidence that shows that attention can be flexibly allocated to representations stored in working memory (in the absence of visual information) was obtained in an fMRI study by Lepsien and Nobre [23]. In a match to-sample task, on each trial, participants were shown in the center of the display either a face or a scene in a sequential order. A double retro-cueing procedure followed the stimuli instructing the participants whether to focus attention on the presented face or scene in order to perform a match-to-sample task. BOLD signals were obtained at the fusiform gyrus (FG) and parahippocampal gyrus (PHG) during the different delay intervals within a trial (following the cues). These areas are known to be modulated by the presentation of faces and scenes respectively (FFA [24], [25]; PPA [26], [27]). The obtained time courses showed increased BOLD amplitudes in the left and right PHG when participants were attending to the memory representation of an initially cued scene-stimulus, but the BOLD levels dropped after the second cue, when it indicated that participants had to switch to the memorized face representation. The reversed effect, but somewhat weaker, was obtained in the right FG, showing an increase in BOLD signal after an initial face-cue followed by a decrease in BOLD signal after the second cue indicated a switch to the memorized scene representation. The results obtained by Lepsien and Nobre [23] show that working memory representations can be enhanced by attention to the memorized features and that the effects of this modulation are coded in visual-cortical areas (i.e., FFA, PPA).

An issue that has been largely unaddressed is whether attention can be shifted to different objects or locations within a working memory representation, similar to the way attention can be shifted from one location to another within a visual scene. That is, are internal working memory representations modulated by attention in the same flexible way as external visual representations are? Given that attentional modulation of neural activity in visual areas can be observed in striate and extrastriate cortex, even in the absence of visual information [28]–[31], the question arises whether allocating attention to locations within a working memory representation modulates lower-level visual areas such as striate and extrastriate cortex in a similar retinotopic way.

In the current study, we investigated whether shifting attention within a working memory representation in the absence of visual stimulation would activate low-level visual cortical areas coding the relevant memorized locations. Participants were asked to memorize the location of four different stimuli, each presented in a separate quadrant of the screen. During the delay period participants were shown a word-cue referring to one of four stimuli. They were instructed to maintain the exact location of the stimulus indicated by the word-cue in working memory. After a second delay a second word-cue was presented which instructed participants to either keep memorizing the same location (half of trials) or to shift their attention to another stimulus and keep that exact location in working memory (another half of trials). At the end of the trial participants saw a black plus-sign (the probe) and had to indicate whether it was presented at the same or a slightly different location than the memorized location. Note that the stimuli were never present during presentation of the cues and selection of to-be-memorized locations occurred on the basis of the memorized representation. If attention enhances working memory representations in a similar way compared to perceptual stimuli, an increase in BOLD activity was expected to be obtained in retinotopically specific regions of the visual cortex that code the location of the stimulus that is being attended in response to the first cue. Once attention shifts towards a different location within the working memory representation, BOLD signals are expected to drop at the initially cued location, whereas a rise in BOLD signal should be observed at the region where attention has shifted towards.

## Materials and Methods

### Participants

Twelve paid volunteers participated in the fMRI experiment. None of the participants reported health problems and all had normal or corrected-to normal eye sight. All reported results are based on data obtained from these twelve participants (3 males, mean age 24.4 years old). The experimental procedure was conducted following the guidelines laid down in the Helsinki Declaration and was approved by the ethical committee of the VU University Medical Center, Amsterdam, The Netherlands. All participants gave written informed consent, prior to the start of the experiment. Participants received either a monetary reward or course credits for taking part in the experiment.

### Stimuli and procedure

The experiment was conducted while participants were lying in the bore of a 1.5T MRI scanner at the VU University Medical Center, Amsterdam. All stimuli were projected on a canvas screen which the participant viewed through a mirror attached to the MRI's head coil. Stimulus presentation and data collection were controlled using E-Prime 1.1 (Psychology Software Tools).

**Figure 1:**
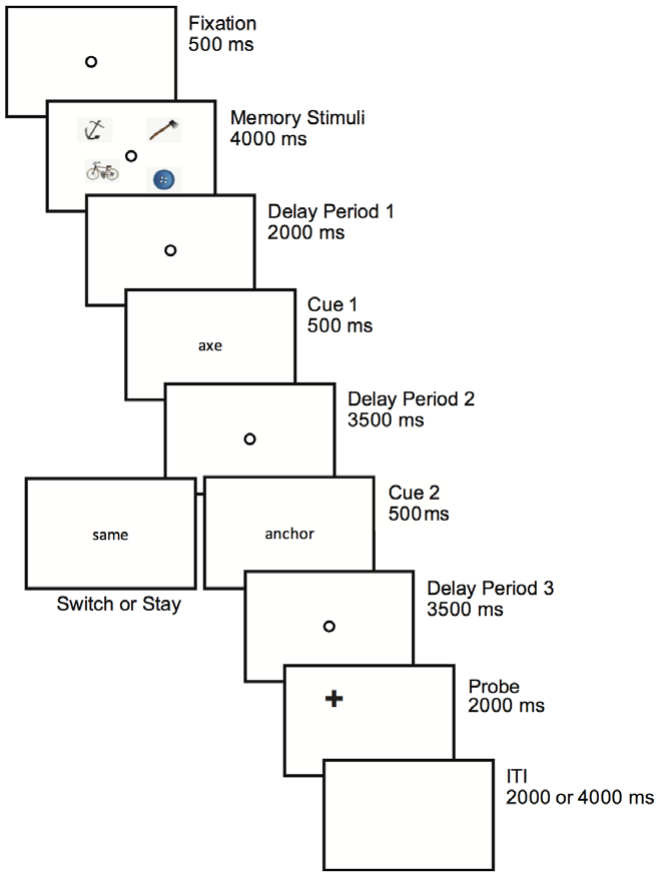
Time course of a typical experimental trial. Participants were presented with four possible target pictures (the memory stimuli) for four seconds, after which they were removed from the screen. This was followed by a 2-second delay period which was used as baseline for the event-related BOLD responses. The first verbal retro-cue (500 ms) instructed participants to remember the exact location of one of the four presented pictures. Four seconds after the onset of the first retro-cue, a second cue was presented consisting of either the word “same” or a word referring to one of the remaining three memory stimuli. This cue instructed participants to either shift attention to the location of another stimulus stored in working memory, or to maintain the location that was already attended. Four seconds after the onset of the second cue a probe was presented and participants indicated whether the probe was presented at the same location as the currently attended item in working memory.

Figure 1 represents the time course of a typical trial. Each trial started with a fixation circle (500 ms) followed by four memory stimuli. These stimuli always consisted of four different objects, randomly sampled from a subset of fourteen object-pictures. The pictures consisted of simple drawn colored objects such as an anchor, a heart or an axe. The four stimuli were simultaneously presented for a period of four seconds, one stimulus per quadrant of the screen. The position of each stimulus was randomly jittered along the imaginary line connecting the center of the picture to the center of the screen, such that on average stimuli were presented at equal distance from fixation (6.14 visual degrees with 0–1.28 degrees random jitter along the diagonal axis) and from each other (horizontally or vertically; average 8.65 visual degrees). To ensure that participants did not focus attention on iconic memory representations of the stimuli, a fixation screen (delay period 1; 2 s) followed the offset of the memory stimuli. Immediately after this, the first retro-cue (Cue1) was presented (500 ms) followed by a fixation screen (delay period 2; 3500 ms). The cue consisted of a word indicating the name of one of the four presented memory stimuli (i.e. “axe”). Participants were instructed to memorize the exact location of the cued stimulus in order to perform a location based delayed-recognition task. Note that the cue by itself did not inform about the memorized location: Participants had to actively remember the location and the identity of the different stimuli in order to be able to use the cues. Four seconds after the onset of Cue1 a second cue would appear (Cue2, 500 ms), defining whether the current trial would be a “switch trial” or a “stay trial”. On 50% of the trials, Cue2 consisted of the word “same”, resulting in a trial in which participants did not have to switch locations as they had to continue maintaining the already selected location in working memory. The word “same” was used so that participants could not rely on Cue2 alone and had to encode Cue1 as well in order to perform each trial properly. On the remaining switch trials, Cue2 consisted of the name of one of the remaining memorized stimuli. Therefore, Cue2 instructed participants to shift attention to the location of another memorized stimulus and to maintain its location in working memory. The switch was always made either horizontally or vertically, but never diagonally. The stimulus presented diagonally opposite to the first cued stimulus would therefore never function as a memory stimulus in that particular trial. A third delay period (3500 ms) was introduced after Cue2 after which the probe stimulus would appear consisting of a black plus-sign. Participants had to indicate whether the probe was presented at the currently memorized location, or whether it was presented at a different location. When the location was the same, participant pressed a button with their left index finger, while for a different location the right index finger was used. The task was unspeeded and accuracy was stressed.

The probe was presented in the cued quadrant on 70% of the trials (i.e. in the quadrant indicated by Cue1 in the stay trials or the quadrant indicated by Cue2 in the switch trials). When the test stimulus was presented within the cued quadrant, it appeared at the exact cued location in 50% of the trials and on the rest of the trials it was slightly displaced from this location (always 1.54° closer to or further away from fixation (chosen randomly) along the diagonal. On 30% of the trials, the probe was presented in a different (non-attended) quadrant, at the location of the stimulus presented in that quadrant. The probe screen would remain onscreen for a period of 2 seconds after which an intertrial interval (ITI) of 2 or 4 seconds (50% each, randomly distributed over conditions) followed, during which the screen was blank. The onset of the fixation circle signaled the beginning of a new trial.

### Scan acquisition

Scanning sessions were performed at the VU University Medical Center using a 1.5 Tesla Siemens Sonata MRI scanner (Siemens Medical Systems, Erlangen, Germany). Functional and structural images of the brain were obtained using an 8-channel phased-array head coil. Whole-brain functional images were collected, using an EchoPlanar Imaging sequence (EPI) with the following parameters: Number of slices = 24, TR = 2000 ms, TE = 83 ms, flip angle = 90°, slice thickness = 4 mm, gap = 0.8 mm, acquisition matrix = 64×64, in-plane resolution = 3.0×3.0 mm. For the retinotopic mapping tasks (Polar and ROI, see section below) EPI-sequences with the following parameters were used (Polar/ROI): number of slices = 22/24, TR = 2290/2000 ms, TE = 104/83 ms, flip angle = 90°, slice thickness = 3.0 mm, gap = 0.6 mm, acquisition matrix = 64×64, in-plane resolution = 3.0×3.0 mm. Each volume was online motion-corrected to reduce artifacts caused by head- or body movements. A whole-brain anatomical 3D image was generated at the end of the scanning session, using a T1-weighted MP-Rage sequence with the following parameters TR = 2730 ms, TE = 3.43, TI = 1000 ms, flip angle = 7°, sagittal slice thickness = 1 mm, acquisition matrix = 256×224 pixels, in-plane resolution = 1×1 mm.

### Retinotopic mapping of visual areas

Two separate tasks were used to map the Regions-of-Interest (ROIs) in visual cortex. These ROIs represented the possible locations of the memory stimuli in the four quadrants of the visual field. First, the borders of visual areas V1, V2 and V3 were mapped using a slowly rotating red/green checkerboard wedge, stimulating the entire visual field. The wedge, having an angle of 30°, performed 20 rotations, each rotation lasting 14 TR (approximately 32 seconds), resulting in a total scan-time of 10.4 minutes. The wedge was presented on a black background.

**Figure 2:**
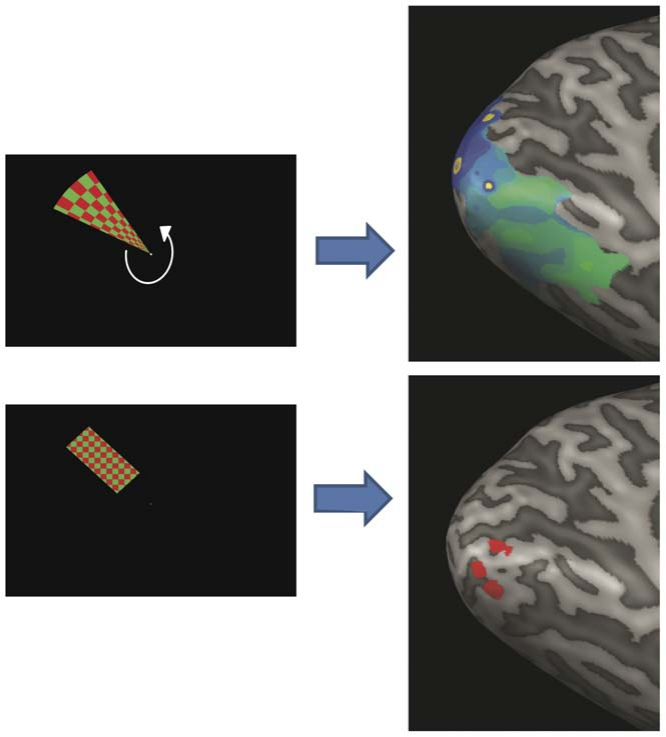
Retinotopic mapping of Regions-Of-Interest (ROIs). In order to define the borders of V1–V3, participants viewed a rotating green and red flickering wedge stimulus. Additionally, a task was used that stimulated the locations of the possible memory stimuli. This was accomplished by presenting a flickering (red/green) rectangle at these locations. This task was used to define the ROIs within V1–V3 for all possible memory locations. The two tasks combined resulted in four ROIs per region of the visual cortex. Event-related time-courses at these ROIs were extracted for the different conditions of the experiment.

Second, to pinpoint the ROIs corresponding to the locations of the memory stimuli, participants were shown rectangles with a red/green checkerboard pattern, counterphased at 9 Hz. The rectangles were presented sequentially and in random order at the four possible memory locations. The rectangles (6.5°×2.85°) were placed diagonally over the axis on which the memory stimuli were presented and covered the entire range of possible memory locations, caused by the spatial jitter of these stimuli. Over two runs, each location was stimulated 40 times in total, each stimulation lasting 2 seconds. After each presentation of a rectangle a random, but equally often presented, ITI of either 2 seconds (1TR) or 4 seconds (2TR) was presented showing only the black background. These two tasks combined allowed the defining of the borders of V1–V3 and the location of the ROIs within these borders (see Figure 2).

### MRI data analyses

All functional data analyses were performed with BrainVoyager QX 2.3 (Brain Innovation, Maastricht, The Netherlands). In order to avoid differences in T1 saturation the first two volumes of each run were discarded. All data was preprocessed in the following manner: Slice scan time correction, spatial smoothing (3 mm FWHM Gaussian kernel), Temporal Filtering (GLM-Fourier: 3 cycles) including linear trend removal. Following preprocessing, the functional data sets of each participant were automatically co-registered in 3 dimensions to the individual anatomical data set. Where necessary this alignment was manually fine-tuned to obtain optimal overlap between functional and anatomical data. Subsequently, all functional data sets, as well as the anatomical data set, were transformed to Talairach space [28], resulting in 4D functional data sets.

By defining the boundary between gray and white matter in the Talairach transformed cortex, a 3D model was created of the cortical surface for both hemispheres of each subject individually. Subsequently, the 3D models were inflated and functional activity from the two localizer tasks were plotted on the inflated hemispheres. Based on this activity the borders between V1, V2 and V3 were defined as well as the four memory locations within each of these areas.

**Figure 3:**
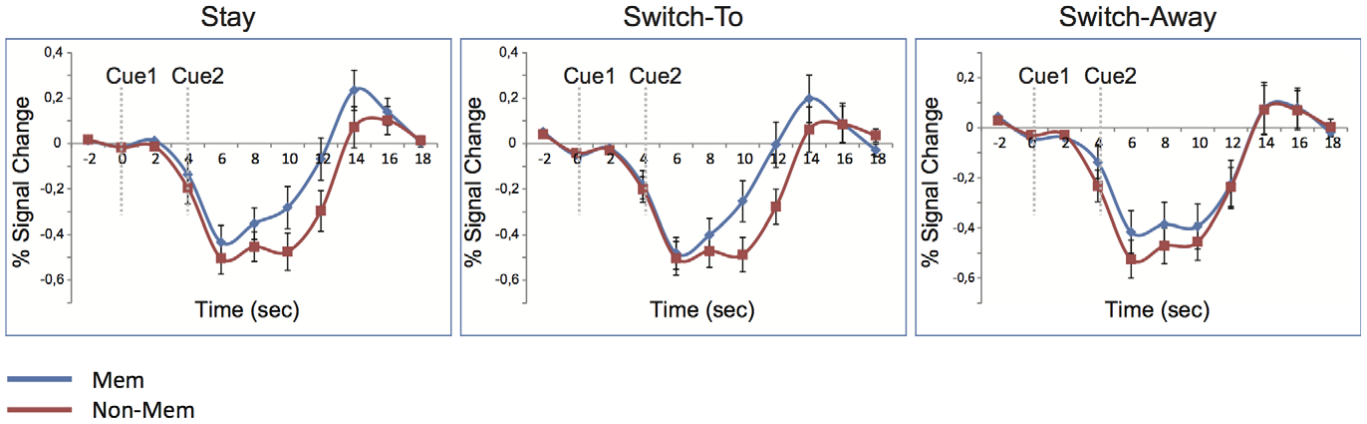
Time courses averaged over V1, V2 and V3 for the different conditions in the experiment. The blue line indicates the BOLD signal obtained at the memory location, whereas the red line indicates the BOLD signal derived from the non-memory locations. The first cue was presented at time point 0, the second cue was presented four seconds later. The time points at 6 s and 8 s reflect the effects of the first cue, whereas the time points at 10 s and 12 s reflect the effects of the second cue. Error-bars represent the standard error of the mean.

Effects of updating spatial working memory content were studied by measuring blood oxygen level dependent (BOLD) responses as obtained by calculating the event-related average independently for each condition, ROI and participant. The 2 second interval prior to the onset of the first cue was used as a baseline for the averages. The used paradigm allowed dividing trials into three different categories (trial-types). First, a location in the visual field, as coded by retinotopically specific ROIs in V1–V3, could contain the memory stimulus for an entire trial when no switching occurred (stay trials). Second, ROIs could code the location of the memory stimulus specified by the second cue, but not by the first cue. In this condition the ROIs did not code the memory location early on in the trial, but based on the second cue participants shifted towards the location coded by these ROIs (switch-to trials). Third, the opposite situation could occur where initially the ROIs coded the memory location early-on in the trial, but after the second cue the participants shifted away from the location coded by the ROIs (switch-away trials). Therefore, the involvement of visual cortex in updating spatial working memory content should be visible in the development of the obtained time courses observed for the three different trial-types. Figure 3a shows the time courses (event-related averages) for the three different trial types, averaged over ROIs. At time point 0, the first cue was presented, whereas the second cue was presented 4 seconds (TR2) after the onset of the first cue. For the purpose of analysis the time period 6–10 seconds after presentation of each cue was taken, as the BOLD reaches its maximal amplitude in this interval. For the current analysis, we look at the interval from 6–10 seconds (early, cue1) and 10–14 seconds (late, cue2).

## Results

### Behavioural data

Mean accuracy for the task was found to be at 77% (*sd* = 6%) correct responses overall. A repeated-measures ANOVA with Trial-type (switch vs. stay) and Probe-location (same quadrant vs. different quadrant) showed no main effect of Trial-type (switch: 78.0%, stay: 75.9% correct; *F*<1).

### fMRI data

The functional MRI data was analyzed according to three different trial types: Stay trials, Switch-to trials and Switch-Away trials. When necessary, Greenhouse-Geisser corrections have been applied.

#### Stay Trials

These trials reflect the situation when the participant had to keep the location of the same object in working memory throughout the whole trial. The left panel of Figure 3 shows the time course of the stay trials averaged for V1–V3. The blue line indicates the averaged BOLD response observed at the cued memory location. The red line indicates the averaged BOLD response over the remaining three non-memory locations. A repeated measures ANOVA with Interval (early vs. late), Location (memory vs. non-memory) and ROI (V1–V3) showed a significant difference in activity between the BOLD responses obtained at the memory location compared to the averaged activity at the non-memory locations (*F*(1,11) = 60.566, *p*<0.001). This differential activation (the difference between BOLD-response obtained at memory compared to the non-memory locations) was found to be present for both early and late intervals (early: *F*(1,11) = 13.832, *p* = 0.003; late: *F*(1,11) = 140.382, *p*<0.001). Although both early and late intervals showed greater activity at the memory location compared to the non-memory location, this difference was found to be larger in the late interval as indicated by an interaction between Location and Interval (*F*(1,11) = 62.931, *p*<0.001).

**Figure 4:**
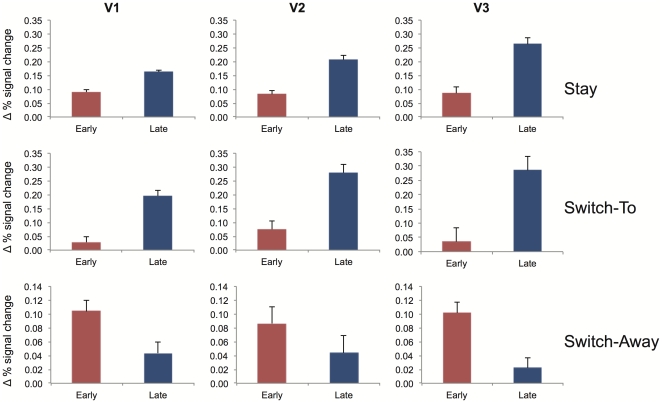
Difference in BOLD signal between memory and non-memory locations (memory – non-memory), separate for each ROI, condition and interval. Error bars indicate the standard error of the mean.

A main effect of ROI (*F*(2,22) = 20.063, *p*<0.001) and a 3-way interaction between ROI x Location x Interval (*F*(2,22) = 4.979, *p* = 0.016) indicated that the difference over time between memory and non-memory locations differed over ROI. The top row of Figure 4 shows the difference between the activity observed at the memory location and the non-memory location separately for the individual ROIs (collapsed over quadrants) and the two different intervals. Post-hoc analysis per ROI exhibited the same effects as the overall analysis for Location and Interval, showing significant differences between the memory and the non-memory location both early and late, for each ROI (V1: early *F*(1,11) = 9.550, *p*<0.010; V1: late *F*(1,11) = 71.555, *p*<0.001; V2: early *F*(1,11) = 11.151, *p* = 0.007; V2 late *F*(1,11) = 66.534, *p*<0.001; V3: early *F*(1,11) = 7.024, *p* = 0.023; V3: late *F*(1,11) = 44.078, *p*<0.001). For each ROI the late interval showed significantly greater differential activity compared to the early interval as indicated by interactions between Location x Interval (V1: *F*(1,11) = 19.238, *p* = 0.001; V2: *F*(1,11) = 27.145; *p*<0.001; V3: *F*(1,11) = 31.881, *p*<0.001).

These results indicate that when participants kept attending to the location of the same stimulus in working memory throughout a trial, significantly stronger BOLD signal was observed at the ROI coding the attended memory location compared to the non-attended locations. This effect increases over time.

#### Switch-to trials

In switch-to trials the location coded by an ROI was not attended initially, but was attended and kept in memory after participants switched towards this location based on the second cue. The middle panel of Figure 3 shows the time course of the switch-to trials averaged for V1–V3. The blue line indicates activity obtained at the memory location, whereas the red line indicates activity obtained at the location that is diagonally opposite towards the memory location. This non-memory location was chosen because participants never shifted diagonally, making this location a valid non-memory location. A repeated measures ANOVA with Interval (early vs. late), Location (memory vs. non-memory) and ROI (V1–V3) showed a significant interaction between Interval x Location, displaying larger differential activation for the late interval compared to the early interval (*F*(1,11) = 54.395, *p*<0.001). Post-hoc tests showed that the difference between the BOLD responses obtained at the memory and non-memory location was significant both in the early and late interval (early: *F*(1,11) = 5.633, *p* = 0.037; late: *F*(1,11) = 106.391, *p*<0.001).

The middle row in Figure 4 shows the differential activation for the switch-to trials for the individual ROIs and intervals. Planned comparisons showed an interaction between Interval x Location for each ROI (V1: *F*(1,11) = 32.100, *p*<0.001; V2: *F*(1,11) = 34.279, *p*<0.001; V3: *F*(1,11) = 30.504, *p*<0.001). Each of these interactions was caused by a significant difference between the memory and non-memory locations during the late interval, when the ROI was coding the memory location, and a non-significant difference during the early interval, when a different ROI was coding the memory location (V1: early *F*<1; V1: late *F*(1,11) = 30.507, *p*<0.001; V2: early *F*(1,11) = 3.437, *p* = 0.091; V2: late *F*(1,11) = 31.124, *p*<0.001; V3: early *F*<1; V3: late *F*(1,11) = 56.025, *p*<0.001).

The results obtained during switch-to trials indicate an increase in differential BOLD activity at the ROIs coding the location that attention switched towards. This differential activity observed at the later stages of the trial when attention was focused at the memory location was larger compared to the early interval (at the ROI level), when attention was focused elsewhere.

#### Switch-away trials

Contrary to the switch-to trials, switch-away trials reflect those trials in which an ROI initially codes the memorized location, but stops doing so after participants switch attention away from it as a result of the second cue. Similar to the switch-to trials, effects of this switch should be observed in the time course of activation. The right panel of Figure 3 shows the time course of the switch-away trials averaged over V1–V3. Again, the blue line indicates the BOLD signal obtained at the memory location, whereas the red line represents activity obtained at the diagonally opposite location. A repeated measures ANOVA with Interval (early vs. late), Location (memory vs. non-memory) and ROI (V1–V3) resulted in a significant interaction between Interval x Location, showing larger differential activation for the early interval compared to the late interval (*F*(1,11) = 8.880, *p* = 0.013).

Planned comparisons showed that the difference between the BOLD responses obtained at the memory and non-memory location was only significant in the early interval and not in the late interval (early: *F*(1,11) = 8.392, *p* = 0.015; late: *F*(1,11) = 1.100, *p*<0.317). The bottom row of Figure 4 shows the differential activation for switch-away trials for the individual ROIs and intervals. Analysis per ROI showed significant interactions between Location x Interval for V1 and V3 (V1: *F*(1,11) = 5.395, *p*<0.040, V3: *F*(1,11) = 10.150, *p* = 0.009), indicating larger differential activation in the early interval compared to the late interval. Although the results obtained at V2 showed a numerically similar pattern, the interaction between Location x Interval did not reach significance in this region (V2: *F*(1,11) = 1.671, *p*<0.223). Additional planned comparisons showed that the difference between the BOLD responses obtained at the memory and the non-memory location did not reach significance in any of the ROIs for the late interval (V1: *F*(1,11) = 1.208, *p*<0.295, V2: F<1, V3: F<1). For the early interval the effects were marginally significant in V1 and V2 (V1: *F*(1,11) = 3.613, *p*<0.084; V2 *F*(1,11) = 4.662, *p*<0.054) and fully significant in V3 (*F*(1,11) = 5.937, *p* = 0.033).

Changes in BOLD signal obtained during the switch-away trials showed the reversed pattern when compared to the switch-to trials. Early on in the trial, when participants focused on the location in working memory coded by the ROI, significant differential activation was observed, whereas this difference disappears when participants shifted attention away from this location towards another location.

## Discussion

The current study investigated the neural correlates of allocating and shifting attention within a working memory representation. The results of this study show that modulation of the BOLD signal corresponds to the attended location currently active in working memory. Changes in BOLD activity were observed in regions of early visual cortex (area V1, V2 and V3). The allocation of attention to stimuli in working memory occurred in the absence of visual stimulation. As a consequence, when participants switched attention away from a cued location, the time course obtained at the ROI coding this location showed differential activation early on in the trial followed by reduced differential activation during the later stages of the trial. The reversed pattern was obtained when participants switched attention towards a location. In this condition, time courses obtained at the ROI showed little or no differential activation in the early stages of the trial and increased differential activation later on. When participants did not have to switch attention between locations within the working memory representation, there was sustained differential activation at the ROI coding the memory location throughout the whole trial.

In both switch-to and switch-away trials changes in BOLD signal over time reflect the crucial findings of this study, showing that changes in BOLD signal follow shifts of attention within the working memory representation. In addition, an effect over time was observed during the stay-trials as well, showing greater differential activation in the later stages of the trial. In theory, no differences in differential activation were expected to be observed in the “stay” condition. The effect obtained during stay trials may be inherent to the used experimental design in which the second cue is more essential for the extent to which a location is being actively maintained in working memory. After the first cue, participants still had to switch attention to a new location on half the trials. This means that participants had to maintain an active representation of the uncued locations of the remaining memory stimuli as well. Once the second cue had appeared this was no longer the case. After the second cue, participants only had to memorize the location of the representation specified by the second cue. The representations of the other non-cued stimuli no longer had to be maintained. As a result, the allocation of attention to the location of the item indicated by the second cue most likely lead to a decay of the representation of the now unattended and irrelevant stimuli [29].

The idea that participants maintain a representation of all stimulus locations in working memory, prior to the onset of the second cue, is supported by the finding that an early overall (averaged over ROIs) memory effect is observed for all three trial types. Thus, even in the switch-to condition, in which participants initially should not attend the location under scrutiny, a memory effect is still obtained over ROIs coding the location where the participant will switch to in the second part of the trial. Additionally, this may also explain why the overall effects in switch-to and stay trials appear to be very similar. As initially to a certain extent, all stimuli are being held in working memory, an early overall effect is observed in the switch-to condition similar to the stay (and switch-away) conditions. Once the second, definitive cue appears, both the switch-to and stay location show increases in differential activity, whereas the pattern of differential activity obtained at the ROI coding the location from which the participants switch-away decreases.

Figure 3 shows that the obtained BOLD responses have an overall negative value. The negative nature of the obtained BOLD responses does not necessarily reflect reductions in blood flow as has been hypothesized [30], but is most likely caused by the choice of the baseline interval in the current experiment combined with the use of an event-related averaging procedure. The current baseline was chosen as the 2 second interval prior to presentation of the first cue (delay period 1) directly following the offset of the memory stimuli. Undoubtedly, due to the sluggishness of the BOLD response some residual processing of the visually presented stimuli was still taking place during the baseline period, making the current baseline not a neutral one that reflects the average neural response when no specific visual information is being processed. Rather, the baseline is most likely elevated above these average levels due to residual visual processing. Consequently, in the later stages of the trial when the BOLD signal caused by visual processing is extinguished, overall activation levels drop, resulting in a negative BOLD-response due to the absence of visual stimulation. Importantly, the differential activity obtained in this study is independent of the effects of the chosen baseline.

Even though the current results show modulation of visual cortical activity as a result of allocating attention to working memory representations, visual cortex is not the only part of the cortex involved in this type of attentional processing. In a recent fMRI study by Nobre et al. [21], using a match-to-sample task, participants were informed about the location of a memory stimulus by either using a pre-cue or a retro-cue. In the pre-cue condition attention could act upon the perceptual representation of the memory stimulus, whereas in the second condition participants' attention was guided to the memory representation of this stimulus. Cue induced patterns of activity for both conditions were observed in mid- and high-level visual areas as well as an indication that V1 might be involved in allocating attention to working memory representations as well. (Observed activity in V1 did not reach statistical significance when correcting for multiple comparisons. Nonetheless, the study by Nobre et al. provides additional evidence that not only high-level visual regions are involved in the allocation of attention to working memory representations, but that lower-level visual cortex is involved as well.) Additionally, a strong overlap in frontal and parietal regions was observed, confirming the role of these areas in spatial working memory and spatial attention. Furthermore, activity in a number of prefrontal regions was selectively observed for orienting attention towards locations actively maintained in working memory. These regions, amongst which the pre-SMA and right middle frontal gyrus, have been shown to be involved in working memory tasks before [31], [32]. The study by Nobre and colleagues shows that focusing attention on locations within internal representations largely draws on the same neural mechanisms used in allocating attention towards external perceptual representations. Combined with the finding that no behavioral differences were observed between pre-cued and retro-cued trials, it seems a valid assumption that the mechanism underlying the two types of attentional allocation is highly similar.

It is important to note that we did not use arrow cues to guide attention to one of the locations. Previous research has shown that arrow cues may automatically guide attention to a location in space [33]. The presentation of an arrow cue may therefore not necessarily represent the allocation of attention to a memory representation but rather the allocation of attention to the external physical display. The present study used verbal cues which need to be processed and interpreted in order to direct attention to the appropriate location. It is therefore unlikely that cues that were used resulted in the allocation of attention to the physical visual environment, as may have been the case in previous studies (e.g., [21]).

The overlap between attentional allocation to external perceptual representations and internal working memory representations has been observed in studies employing EEG. Kuo et al. [18] showed that searching for a target elicited similar N2pc components irrespective of whether participants were searching for a target in a perceptual array of stimuli or whether this array was maintained in working memory. The N2pc component is strongly associated with attentional selection, both reflecting target enhancement as well as distractor suppression [34]. Although the N2pc is thought to be generated at occipital-temporal and posterior-parietal parts of the brain and not in low-level visual cortex, this study does emphasize the involvement of visual areas in allocating attention to working memory representations.

The contribution of visual cortex to visual-spatial working memory is in line with current models that claim that working memory maintenance does not primarily take place in prefrontal cortex but is mediated by posterior visual areas. Postle [35] claimed that working memory is an emergent property of neural processes required for perception and action, mediated by an attentional mechanism acting upon the cortical regions responsible for these neural processes. More specifically, the framework proposed by Postle and colleagues states that spatial working memory is not dependent on a specialized mnemonic mechanism such as the visuo-spatial sketchpad, but can be derived from spatial selective attention (and motor control functions), as observed in Awh's rehearsal theory [36], [37]. In general, the model proposes a role for neural regions that are responsible for sensory and action related processing in working memory, without there being a separate memory mechanism for different types of working memory tasks. The role of the prefrontal cortex in this model is not one of storage of information; rather it is hypothesized to be involved in control processes such as filtering of irrelevant information [38] or attentional monitoring and selection [39].

Although an ongoing debate concerning the specific contribution of frontal, parietal and visual areas to the maintenance of working memory representations remains, the current study shows that coding the location of an item stored in working memory results in retinotopically specific changes in BOLD-signal in early visual cortex, including V1. Importantly, attention can be shifted between locations stored in working memory, which in turn leads to spatially specific modulation of only those visual areas that code the attended (switched to) location. The pattern of differential activation remains significant during the delay period between cue and probe as long as this region is relevant to the task. When an ROI is no longer coding the relevant and attended location, differential activation obtained at these ROIs decreased. Similar results have been obtained by studying working memory processes with single-cell recording in monkeys [40].

The notion of visual working memory is closely related to the concept of visual mental imagery, both conceptually and in terms of neural correlates [41], [42]. Mental imagery has often been referred to as seeing in the absence of visual stimulation [43] or “seeing with the mind's eye” [44], [45], referring to the ability of vividly reactivating previously experienced visual percepts such as objects or object properties as well as spatial relations between these objects [16]. In addition, the envisioned mental images can be the result of combining or elaborating on one or multiple previously perceived visual representations, resulting in novel mental images [46].

In terms of neural correlates, a significant amount of overlap in neural structures supporting both constructs has been observed in the past decades. Similar to visual working memory, involvement of frontal and parietal control areas has been observed in visual mental imagery [41], [47], [48], as well as the involvement of sensory occipital areas [49], [50]. Regardless of the broad overlap between cortical areas involved in visual working memory and visual imagery, the neural correlates of these constructs do dissociate. Neuroimaging studies have shown that distinct areas can be activated by working memory and imagery along with differential neural activity in cortical regions that do overlap [41].

Nevertheless, the extended overlap between visual imagery and visual memory complicates the prying apart of these two constructs, especially in experimental designs that solely attempt to measure working memory processes. Therefore, one can wonder how the observed effects in the current study relate to visual mental imagery. Are the effects observed in early visual cortex the result of imagery rather than visual working memory? The answers to these questions are not straightforward, yet two lines of prior research suggest that the current data may indeed reflect working memory processes.

First, Baddeley has hypothesized that visual mental imagery may well depend on the visual-spatial sketchpad [51] (but see [35]), one of the slave-systems often mentioned in classic studies of working memory [52]. The use of the visual-spatial sketchpad for mental imagery may very well mean that visual working memory relies on imagery at least to some extent. Therefore visual imagery and visual working memory may not necessarily represent the same construct but are often found to be inseparably intertwined. This may entail that the current results do not allow a clear distinction between the two concepts.

Second, and more importantly, a number of studies have shown clear effects of mental imagery in early visual areas [49], [50]. It has been shown that these effects are most prominent when participants were instructed to perform a task which did not require spatial judgments to be made (but for example a shape evaluation was required). When a spatial judgment task had to be performed based on imagery, no effects in early visual areas were present (for an overview, see [46]). Although not definitive, it seems that making spatial judgments within representations of visual mental imagery may not draw upon these early visual areas. As the current study shows clear effects in V1–V3, it can therefore be argued that the results in the current task may not rely on mental imagery, but may reflect distinctive visual working memory processes.

The current study provides insight in the way attention can be shifted within working memory representations in a flexible, top-down manner. Similar to real life situations, spatial attention is utilized in order to facilitate selection and perception of objects or locations that are relevant for every day behavior. The current study shows that this flexible attentional mechanism does not merely act upon external perceptual representations, but also on stored working memory representations in the same dynamic way. In addition, this study shows that the earliest regions in visual cortex are modulated by allocating attentional resources to working memory representations. To summarize, this study shows that attention can be shifted between locations within a working memory representation which leads to modulation of retinotopically specific areas of low-level visual cortex, dependent on where attention is focused.
